# Spatial pattern of body mass index among adults in the diabetes study of Northern California (DISTANCE)

**DOI:** 10.1186/1476-072X-13-48

**Published:** 2014-12-04

**Authors:** Barbara A Laraia, Samuel D Blanchard, Andrew J Karter, Jessica C Jones-Smith, Margaret Warton, Ellen Kersten, Michael Jerrett, Howard H Moffet, Nancy Adler, Dean Schillinger, Maggi Kelly

**Affiliations:** School of Public Health, Division of Community Health and Child Development, University of California, 207-B University Hall, #7360, Berkeley, CA 94720-7360 USA; Department of Environmental Science, Policy and Management, College of Natural Resources, University of California, Berkeley, Berkeley, CA USA; Kaiser Permanente Division of Research, 2000 Broadway, Oakland, CA 94612 USA; Department of International Health, Division of Human Nutrition, Johns Hopkins Bloomberg School of Public Health, Baltimore, MD USA; Division of Environmental Health Sciences, School of Public Health, University of California, Berkeley, Berkeley, CA USA; Department of Psychiatry, University of California, San Francisco, USA; Department of Medicine, University of California, San Francisco, USA; Department of Environmental Science, Policy and Management, Ecosystem Sciences Division, University of California, Berkeley, Berkeley, CA USA

**Keywords:** Body mass index, Diabetes, Spatial clustering, Moran’s I, Spatial autocorrelation, Neighborhood characteristics, Geographical epidemiology

## Abstract

**Background:**

The role that environmental factors, such as neighborhood socioeconomics, food, and physical environment, play in the risk of obesity and chronic diseases is not well quantified. Understanding how spatial distribution of disease risk factors overlap with that of environmental (contextual) characteristics may inform health interventions and policies aimed at reducing the environment risk factors. We evaluated the extent to which spatial clustering of extreme body mass index (BMI) values among a large sample of adults with diabetes was explained by individual characteristics and contextual factors.

**Methods:**

We quantified spatial clustering of BMI among 15,854 adults with diabetes from the Diabetes Study of Northern California (DISTANCE) cohort using the Global and Local Moran’s I spatial statistic. As a null model, we assessed the amount of clustering when BMI values were randomly assigned. To evaluate predictors of spatial clustering, we estimated two linear models to estimate BMI residuals. First we included individual factors (demographic and socioeconomic characteristics). Then we added contextual factors (neighborhood deprivation, food environment) that may be associated with BMI. We assessed the amount of clustering that remained using BMI residuals.

**Results:**

Global Moran’s I indicated significant clustering of extreme BMI values; however, after accounting for individual socioeconomic and demographic characteristics, there was no longer significant clustering. Twelve percent of the sample clustered in extreme high or low BMI clusters, whereas, only 2.67% of the sample was clustered when BMI values were randomly assigned. After accounting for individual characteristics, we found clustering of 3.8% while accounting for neighborhood characteristics resulted in 6.0% clustering of BMI. After additional adjustment of neighborhood characteristics, clustering was reduced to 3.4%, effectively accounting for spatial clustering of BMI.

**Conclusions:**

We found substantial clustering of extreme high and low BMI values in Northern California among adults with diabetes. Individual characteristics explained somewhat more of clustering of the BMI values than did neighborhood characteristics. These findings, although cross-sectional, may suggest that selection into neighborhoods as the primary explanation of why individuals with extreme BMI values live close to one another. Further studies are needed to assess causes of extreme BMI clustering, and to identify any community level role to influence behavior change.

**Electronic supplementary material:**

The online version of this article (doi:10.1186/1476-072X-13-48) contains supplementary material, which is available to authorized users.

## Introduction

Area level socioeconomic and food environment factors have been associated in cross-sectional analysis with body mass index (BMI) [1–3], insulin resistance [4], and diabetes incidence [5] independent of individual characteristics. The consistency of cross-sectional associations of neighborhood factors with diet sensitive disease risk factors and chronic disease has led to further investigation of potential causal links that may inform policies and programs to improve neighborhood access to health-promoting resources such as healthful food, e.g. supermarkets, produce vendors, and farmer’s markets. Although demonstrating a causal link between area-level socioeconomic deprivation and diabetes incidence is challenging, a growing body of literature strongly suggests that living in a relatively less deprived area is associated with lower diabetes incidence [5–7]. Cox found a greater incidence of diabetes in deprived neighborhoods surrounded by relatively more deprived neighborhoods [5]. Furthermore, the strength of the relationship between deprivation and diabetes incidence was found to increase over time due to selective immobility [7]. In the US, the Moving to Opportunity for Fair Housing was a demonstration study that randomly assigned families housing vouchers to move from high poverty areas to less deprived neighborhoods [8]. The ten-year follow up found that living in a less deprived neighborhood was associated with a lower percentage of adults with severe obesity and high glycosylated hemoglobin values, even though participants lived in similarly deprived neighborhoods at the ten year mark [6]. These findings provide evidence of a socio-spatial association with diabetes risk factors and prevalence, suggesting residential neighborhood context may indeed have a direct influence on risk factors and incident chronic disease.

Among patients diagnosed with diabetes, weight is viewed as a modifiable risk factor, and patients who are obese are encouraged to decrease weight to better manage and limit disease progression [9]. Weight loss among adults with diabetes is associated with better control of blood sugar levels measured by glycosylated hemoglobin, lower levels of blood pressure, cholesterol, and triglycerides, and slower progression of other co-morbidities (such as loss of eyesight, amputation, and loss of kidney function) [10]. The Look AHEAD (Action for Health in Diabetes) Trial, an intensive lifestyle intervention including diet and physical activity, found significant reductions in weight, systolic and diastolic blood pressure, high-density lipoprotein cholesterol and triglycerides, and improvement in treadmill fitness after four years [11]. These improvements persisted after ten years, however, no difference was seen in the rates of cardiovascular events, the ultimate outcome of the trial [12]. Although weight loss or even weight maintenance is important, several studies have found that once diagnosed with diabetes, lower weight status (BMI < 25) is associated with mortality [13].

Identifying and assessing spatial clustering of extreme health values has been considered a hallmark indication that contextual (area level) exposures have an impact on health outcomes. Alternatively, spatial clustering of health outcomes may be the result of personal choices, conditions and preferences which result in residential selection with those with a given health condition living in the same area. For example, poverty may relegate some people to one neighborhood while preference for parks or schools might influence others to live near these resources. Poverty and personal preference may therefore be causally linked with an outcome and not neighborhood attributes. Spatial analysis of health has increased over the past decade, but most public health and epidemiology research is still “aspatial” despite having a focus on place-based influences on health determinants and outcomes [14]. In this study, we sought to understand the extent of spatial clustering of extreme high and low BMI values among a cohort of adults with diabetes and to identify individual- and environmental-level indicators that explain spatial clustering.

Spatial hot spots of adults with diabetes in clusters of high or low BMI values might be interpreted as sentinel communities. Such communities could help us understand what environmental cues promote high and low BMI clusters—either by drawing individuals into the community or by assisting individuals to maintain extreme high or low BMI status. Identifying neighborhoods with high BMI clusters could help direct the distribution of resources for obesity prevention and treatment programs among adults with diabetes, and may “shape an appropriate intervention program tailored for the residents in a particular geographic region” [15].

We hypothesized that high or low BMI values in a cohort of adults with diabetes would cluster geographically. More specifically, individuals with high (or low) BMI values would live close to others with a high (or low) BMI, respectively. We conducted a study among adults with diabetes from the Diabetes Study of Northern California (DISTANCE) to evaluate these hypotheses. Our aims were to: (1) examine the extent to which extreme BMI values were spatially clustered, and (2) identify significant associations between area level factors and spatial clustering in a large cohort of adults with diabetes.

## Methods

### Study population

The Kaiser Permanente Northern California Diabetes Registry was established in 1993 within Kaiser Permanente Northern California, a large, integrated health care delivery system serving more than 3 million members. Kaiser Permanente of Northern California (KPNC) members are 30% of the population of Northern California [16] and have similar demographic and socioeconomic distributions to the population from the surrounding geographical region except for the very extremes of the income distribution; fewer very rich and fewer very poor [16, 17]. The Diabetes Study of Northern California (DISTANCE) was a survey follow-up cohort study. The study subjects were an ethnically stratified, random sample of 40,735 Diabetes Registry members. 20,188 persons completed the survey from May 2005 through December 2006. Moffet et al. [18] provided detailed information on the DISTANCE cohort profile. The analysis sample included respondents who had accurate address information geocoded to street address and matched to the census block (n = 18,962) and who had complete data on BMI and individual variables (n = 15,887). We removed outliers (BMI <18 & >70) which resulted in the final sample of 15,854. Participant’s home addresses were geocoded by Kaiser Permanente at the 2000 census block (street) level using MapInfo’s MapMarker (Pitney Bowes, Stamford, CT), and the latitude and longitude of the census block centroid was used as the participant’s residential point location. The finest geocode level made available to the authors was at the census block. Coincident points (19% of the total sample) were offset from one another using a random offset distance that was constricted to a participant’s census block. The offset is intended to represent a more realistic dispersion of residential addresses within census blocks with multiple participants. The majority (96%) of participant census blocks have an area less than 1 km^2^ (0.38 mi^2^) where 78% of those participant census blocks are under 0.1 km^2^ (0.038 mi^2^). The study area covers all 19 counties in the Northern California Kaiser Permanente service area totaling 70,585 km^2^ (27,253 mi^2^).

### Geospatial approach

Our initial goal was to examine the extent to which extreme BMI values displayed positive spatial autocorrelation. There are a variety of methods to measure spatial autocorrelation which are generally grouped into two categories: (1) global indicators which measure the overall global or population level spatial autocorrelation in a dataset, and (2) local indicators, also known as Local Indicators of Spatial Association (LISA), which measure the spatial autocorrelation of each feature in relation to each neighboring feature in a dataset. Global Moran’s I was selected for this study because it measures overall spatial autocorrelation based on feature attribute values and is intended for datasets where both high and low value clusters are assumed to exist. Additionally, Global Moran’s I is more sensitive to extreme values than similar indicators such as Geary’s C, while Kulldorff spatial scan, Ripley’s K, and Cuzick-Edwards k-Nearest Neighbor indicators do not consider the attribute values of the features under analysis, only their spatial location. Global Moran’s I calculates each feature’s measured index, expected index, variance, z-score, and p-value and indicates if the outcome is overall clustered (positive), dispersed or regular pattern (negative), or randomly distributed over space (zero) [19]. For a description of the use of spatial autocorrelation methods and a detailed table summarizing a selection of spatial clustering methods reported in the public health literature see Additional file 1 and Additional file 2 that accompanies this article.

Local Moran’s I was also selected for this study because it allows for the identification of spatial hotspots of local areas that represent clusters of unexpectedly high or low values compared to the global mean [20, 21] This eliminates any potential bias an extremely high or low value target feature would have in the calculation of its neighborhood mean and is why Local Moran’s I was chosen over other local indicators such as Getis-Ord Gi*. The key difference between Global and Local Moran’s I is that the global index assesses the general tendency for high values to be located adjacent to high values and vice versa across the entire spatial domain to generate one summarized measure. Clusters of extreme BMIs reflect outliers in the population that are greater than two standard deviations from the mean and that are also spatially autocorrelated. Features are then assigned as belonging to either a low/low or high/high cluster of similar feature values based on their value and statistical significance at a 95% confidence level [22]. In our case, cohort patients that were identified as a high/high BMI cluster will themselves have a high BMI, in comparison to the population average, and will be surrounded by other cohort patients that have a similarly high BMI.

### Outcomes

We assessed body mass index (BMI = kilograms/meters squared) and BMI residuals as continuous measures. BMI was calculated from electronic records using the first clinical measurement of height and weight recorded in an outpatient visit within one year before or after the survey date. For individuals with no measured weight and height within two years after the survey, self- reported weight and height from the survey was used (n = 1,226). To produce BMI residuals, multivariate linear regression (ordinary least squares) was performed and BMI was regressed on a set of hypothesized confounders of the relation between BMI and place of residence. Model 1 included individual demographic and socioeconomic factors and Model 2 included neighborhood level factors. BMI residuals represent the portion of BMI not explained by covariates in a model, and the spatial analysis of the BMI residuals can be interpreted as clustering of unexplained low/low or high/high BMI variation [19].

**Model 1** regressed BMI on individual level characteristics that included race/ethnicity (White non-Latino, African American, Latino, Asian, or other), marital status (married/living together, divorced/separated, widowed, never married, or refused/don’t know/missing), sex, age (30-51, 52-64, ≥65 years), education (no high school degree, high school/GED/technical school, associate degree, college graduate, or post graduate education), nativity (number of years in the US and US born), and income to poverty ratio, defined as self-reported family income for a given age and household size divided by the 2005 poverty level income for the same age and household size; this variable was categorized as >600% , 301-600%, 101-300%, 0-100% of poverty level, and “don’t know/refused/missing” [23]. Additionally, we tested if the income to poverty ratio relationship with BMI varied by race and retained the interaction term in the model if it was significant at the α ≤ 0.05 level.

**Model 2** regressed BMI on a set of neighborhood level characteristics that included neighborhood deprivation index (NDI) as a continuous and categorical quartile variable, healthful and unhealthful food environment retail density measures, 2000 census tract population density per square mile and percent of population white, distance to nearest Kaiser Permanente healthcare facility, and 2006 municipal level property and violent crime per 100,000 population rate from the US Federal Bureau of Investigation Uniform Crime Reports. The neighborhood deprivation index (NDI) [24] was created based on 2000 US Housing and Population Census data for the 19 counties in our study area using principal components analysis. We used 2006 food retail data from Dun and Bradstreet’s National Establishment Time-Series (NETS) database [25] to create measures of healthful and unhealthful food retail environments. Food retail data representing the following four categories: supermarkets, produce vendors, convenience stores, and fast food restaurants, were extracted from the database based on Standardized Industrial Codes (SIC). Addresses were geocoded using ArcGIS (ESRI Inc., Redlands, CA). The geocoded point data from each of the four food retail categories were transformed into four distinct continuous raster surfaces representing their respective food retail densities using a kernel density method based on Silverman [26] as implemented in ArcGIS v.10.1 (ESRI Inc., Redlands, CA). Kernel densities of food retail data have been used in neighborhood-health literature to characterize the food environment and provide an estimate of the accessibility or exposure of a population to healthful and unhealthful food outlets [3, 27, 28].

**Model 3** regressed BMI on the above set of individual and neighborhood level characteristics. Additional models were tested that included health indicators of Charlson-Deyo comorbidity score [29], smoking status, and insurance coverage group (MediCal, Kaiser group or Kaiser individual), and a final model added spatial location characteristics including latitude and longitude and latitude and longitude squared and cubed. Spatial location characteristics were used in the model in order to control for spatial autocorrelation. A robust variance estimator was used to account for correlation at the census block level [30, 31]. The analysis was conducted with Stata 12.0 (StataCorp LP, College Station, TX).

### Statistical analysis

#### Spatial autocorrelation analysis

The degree of clustering of BMI and BMI residual values from each regression model for the population (n = 15,854) was conducted using Global and local Moran’s I. Global Moran’s I was calculated using a Euclidian neighborhood search radius of 1.6 km (1 mi) where the target feature (e.g. geocoded address) was weighted at one and the weight of all neighboring features (e.g. neighboring geocoded addresses) within this radius decreased by distance until the 1.6 km threshold was reached. Neighboring features outside the radius are weighted at 0. The 1.6 km radius approximates a typical neighborhood size in our study area and has been found to be associated with health outcomes [32]. See ESRI [22] for a description of the equation for the Global Moran’s I statistic. Similar to Global Moran’s I, Local Moran’s I was calculated using a Euclidian neighborhood search radius of 1.6 km.

We conducted two sensitivity analyses. First, we repeated the calculation of Local Moran’s I using a 1.6 km radius on 100 sets of randomly assigned cohort BMI values. The results were summarized to establish the magnitude and locations of clustering that could exist at random within our study area and to generate bootstrap confidence intervals to aid in interpreting the robustness of the non-randomized Local Moran’s I results. Second, we conducted a sensitivity analysis using a 3.2 km (2 mi) radius to test the sensitivity of clustering to our selected neighborhood radius. Spatial clustering analyses were conducted with ArcGIS v.10.1 (ESRI, Inc., Redlands, CA).

The point feature low/low and high/high cluster spatial results of the BMI, BMI residuals, and one randomly distributed BMI run example were transformed into raster density surfaces for display. The density surfaces perform two functions: first to mask individual locations by smoothing distinct point locations over a larger area and second, to facilitate the identification of spatial patterns within the study area with large concentrations of low/low and high/high clusters. The outcome density surfaces were created using a neighborhood radius of 3.2 km and a cell size of 500 m in units of square kilometers. The resulting density surface depicts a magnitude of the number of points (e.g. geocoded addresses) per unit area that are within the neighborhood radius.

The spatial point results of the cluster analysis for BMI and BMI residuals were examined for patterns in the geographic distribution of any remaining clusters. A visual inspection for geographic locations that had large numbers of BMI residual clusters (≥50) within a municipality and whose location and cluster type persisted throughout each Local Moran’s I BMI and BMI residual model spatial result were noted.

## Results

The race-stratified sample was comprised of 27.1% Asians, 23.4% white, 19.1% Hispanics, 17.8% African-Americans, and 12.7% of other or mixed race. BMI for our sample population ranged from 18.0 to 68.7 with a mean of 31.1 (SD 6.5) (Table 1).

**Table 1:** **Baseline socio-demographic characteristics of study population (n = 15,854)**

Variable	Number	Percent
**Age (years)**		
30-51	3,794	23.93
52-64	6,941	43.78
≥ 65	5,119	32.29
**Sex**		
Female	7,799	49.19
Male	8,055	50.81
**Race/ethnicity**		
White non-Latino	3,708	23.39
African American	2,818	17.77
Latino	3,023	19.07
Asian	4,298	27.11
Other*	2,007	12.66
Income to poverty ratio**		
> 600% poverty level	2,691	16.97
301-600%	4,526	28.55
101-300%	4,074	25.7
0-100%	1,391	8.77
Missing	3,172	20.01
**Marital status**		
Married	10,941	69.01
Living Together	370	2.33
Divorced/Separated	1,900	11.98
Widowed	1,265	7.98
Never married	1,338	8.44
Missing	40	0.25
**Education**		
No high school degree	2,437	15.37
High school/GED/technical school diploma	6,620	41.76
Associate degree	1,800	11.35
College graduate	3,185	20.09
Post graduate	1,537	9.69
Missing education	275	1.73
**Nativity**		
Born in USA	9,901	62.45
Born outside USA	5,930	37.4
Missing nativity	23	0.14

*Other race/ethnicity category includes Pacific Islander, American Indian/Native American, and Alaskan Native.

**Poverty level defined as self-reported family income for a given age and household size divided by the 2005 poverty level income for the same age and household size.

The Global Moran’s I statistic of 0.05 and a z-score of 7.72 indicated that *BMI had a low to moderate level of global autocorrelation* (Table 2). After controlling for individual level factors, the Global Moran’s I statistic for BMI residuals decreased to -0.01, indicating a general random global spatial distribution and suggesting that individual characteristics (Model 1) accounted for spatial autocorrelation of observations. Controlling for only environmental characteristics (Model 2) decreased the Global Moran’s I statistic to 0.02, and it remained significant.

**Table 2:** **Summary of Global Moran’s I cluster analysis results (n = 15,854)**

Analysis Input Value	Moran’s Index	z-score	p-value
BMI	0.05	7.72	0.00
*Confounder Regression Model BMI Residuals*			
Model 1^a^	−0.01	−0.76	0.45
Model 2^b^	0.02	2.63	0.01
Model 3^a,b^	−0.01	−1.11	0.27

^a^controlled for age, education, race/ethnicity, marital status, sex, nativity, income to poverty ratio, and an interaction term for income to poverty ratio*BMI and income to poverty ratio*race/ethnicity.

^b^controlled for food environment, neighborhood deprivation index, percent of population who were white, population density, distance to Kaiser Permanente healthcare facility, and property and violent crime rate.

**Figure 1:**
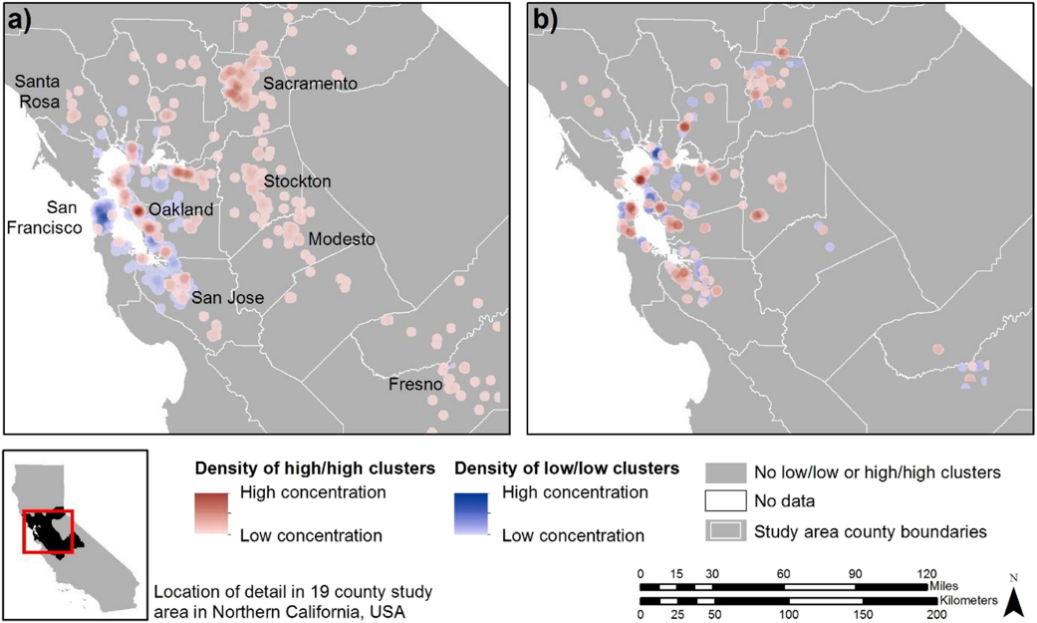
**Spatial clustering of BMI and randomly distributed BMI as a density surface: (a)** Density of low/low and high/high clusters for BMI with major population centers labeled; **(b)** Density of low/low and high/high clusters from one randomized BMI run.

The Local Moran’s I statistic, using a 1.6 km (1 mi) radius, indicated 11.9% of cohort patients are significantly clustered in either a low/low (6.7%) or a high/high (5.2%) BMI cluster (Table 3). Patients in a low/low cluster (n = 1,066) had a mean BMI of 24.2 (range: 18.0 - 29.0, SD: 2.2) and are represented as rasterized circles in blue, while those in a high/high cluster (n = 821) had a mean BMI of 43.8 (range: 33.0 - 68.6, SD: 6.6) and are represented in red in Figure 1(a). The color gradient (light to dark) indicates the relative density or magnitude (one-to-many) of similar value clusters within a 3.2 km (2 mi) radius. A BMI of 43.8 is class III obesity and considered severely obese (e.g. >35 BMI) [33], indicating the cluster analysis is identifying individuals with clinically meaningful high BMIs. Generally, the western San Francisco Bay Area has more low/low BMI clusters, while higher concentrations of high/high BMI clusters are east of the bay or outside the Bay Area.

**Table 3:** Summary of Local Moran’s I cluster analysis results (n = 15,854)*

	Cluster Types			
Analysis Input Value	Low/Low	High/High	Non-Clustered	% Total Clustering
	n (%)	n (%)	n (%)	
BMI	1066 (6.72)	821 (5.18)	13152 (82.96)	11.90
*BMI Residuals*				
Model 1^a^	201 (1.27)	403 (2.54)	14723 (92.87)	3.81
Model 2^b^	365 (2.30)	582 (3.67)	14288 (90.12)	5.97
Model 3^a,b^	186 (1.17)	361 (2.28)	14765 (93.13)	3.45

*Only low/low and high/high clusters for the 1.6 km (1 mi) radius cluster analysis results are depicted. Sum of low/low, high/high and non-clustered do not sum to 15,854 or 100% because low/high and high/low clusters are omitted from table.

^a^controlled for age, education, race/ethnicity, marital status, sex, nativity, income to poverty ratio, and an interaction term for income to poverty ratio*BMI and income to poverty ratio*race/ethnicity.

^b^controlled for food environment, neighborhood deprivation index, percent of population who were white, population density, distance to Kaiser Permanente healthcare facility, and property and violent crime rate.

After controlling for possible confounders using regression Models 1, 2, and 3, the BMI residuals were predicted and again subjected to the Local Moran’s I analysis. The results of Model 1, controlling for individual characteristics, reduced the percentage of the sample population that was spatially clustered by 68%; from 11.9% to 3.8% (Table 3). Among those clustered, 1.3% were in a low/low and 2.5% in a high/high BMI residual cluster. Model 2 controlled for only neighborhood attributes and reduced the percentage of clustering by roughly half (6.0%). Model 3 controlled for both individual and neighborhood characteristics, and the results were similar to that of Model 1. We adjusted for additional individual health status indicators (comorbidity, smoking, insurance type) and spatial location variables (latitude, longitude, and latitude and longitude squared and cubed), but the results did not further reduce the amount of clustering (data not shown).

**Figure 2:**
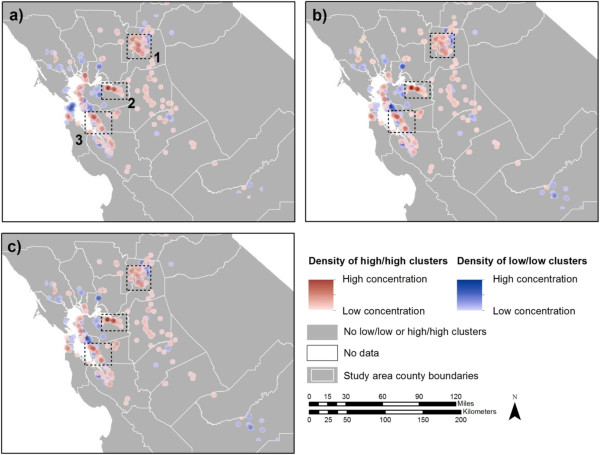
**Spatial clustering BMI residuals as a density surface: (a)** Density of low/low and high/high clusters for BMI residuals from Model 1^a^. **(b)** Density of low/low and high/high clusters for BMI residuals from Model 2^b^. **(c)** Density of low/low and high/high clusters for BMI residuals from Model 3^a,b^; Locations where ≥50 high/high clusters persist in all cluster analysis runs for BMI and both model residuals are highlighted inside three dotted line boxes labeled 1 to 3.

Figure 2 (a), (b) and (c) shows the spatial distribution and density of individuals assigned to low/low and high/high BMI residual clusters from adjusted Models 1, 2, and 3, respectively. Distributions were similar to the concentrations and locations of BMI clusters in Figure 1 (a). While the amount of spatial clustering decreased and some clusters disappeared in the spatial results of BMI clustering after adjusting, adjusting for potential confounders generated no new concentrations of spatial clusters, and the patterns of both low/low and high/high BMI clusters were similar over space.The sensitivity analysis drawing 100 runs of randomly distributed BMI values resulted in 2.67% (95% confidence intervals: 2.61, 2.72) of patients clustered in either in a low/low (mean of 0.8%) or high/high (mean of 1.9%) BMI cluster. Figure 1(b) depicts the density of low/low and high/high clusters from one randomized BMI cluster spatial analysis. The second sensitivity analysis that examined the effect of using a larger distance radius of 3.2 km (2 mi) for Local Moran’s I resulted in an increase in the number of cohort members that were found to be clustered; 16.0% of cohort patients were retained in either a low/low (9.1%) or a high/high (6.9%) BMI cluster (data not shown). The 3.2 km (2 mi) radius cluster analysis of BMI residuals resulted in a similar magnitude reduction of the amount clustered as the 1.6 km (1 mi) radius analysis with a reduction of 72% to 4.5%, with 2.1% in a low/low BMI cluster and 2.4% in a high/high BMI cluster.

Upon visual inspection, three areas with concentrations of ≥50 people persisted. All three areas represented high/high BMI residual clusters (no area had ≥50 of individuals in low/low BMI clusters) and clusters were concentrated within 4.8 km (3 mi) of each other. The number of high/high clusters from Model 3 in the three highlighted areas numbered 76 in area 1, 57 in area 2 and 51 in area 3. These three areas represented roughly 34% (184/547) of the residual group and indicated that high/high BMI residuals among clustered cohort members in these locations were not explained well by our model. The residual clustering may be due to some factor not captured in our models.

## Discussion

This study assessed the presence and extent of clustered low and high BMI values among adult Kaiser Permanente members with diabetes in Northern California. Our findings show a moderately low level of global autocorrelation, but a substantial percent of local clustering of low/low and high/high BMI values. In our case, we applied a 1.6 km (1 mi) radius to determine if local areas have BMI values that are higher or lower than would be expected based on the global average or a random expectation for the entire study area [19]. To directly illustrate this point, we compared the findings to the amount of clustering that would be expected if we randomly assigned BMI values to the sample population 100 times. Comparison of the maps in Figure 1 (observed vs. randomly assigned BMI values) indicates that low and high BMI clusters had different spatial patterns for the majority of the study area. For example, a large number of clusters in the non-randomized BMI results were not found in the randomized BMI results, low BMI clusters in the non-randomized BMI results were reversed and became high BMI clusters in the randomized BMI results, and the magnitude of BMI clustering for both low and high BMI clusters changed between the non-random and random BMI results. This suggests that neither the underlying population distribution of the cohort members nor population density were major factors driving the cluster analysis results.

We found that after adjusting for individual demographic and socioeconomic characteristics the Global Moran’s I was reduced to near zero, suggesting that individual factors accounted for most of the spatial autocorrelation in the BMI values and individual factors explained roughly 68% of the local BMI clustering. Adjusting for only neighborhood factors reduced the Global Moran’s I by half but it remained significant. The pattern of spatial clustering was similar between Models 1 and 2. Although the neighborhood characteristics did not explain as much of the spatial clustering as did the individual factors, 50% and 68% reduction might not represent a substantial difference in regards to spatial clustering of extreme BMI values. Model 3, which accounted for both individual and neighborhood characteristics, reduced clustering the greatest amount, suggesting that the individual and neighborhood factors address spatial autocorrelation and explained almost all of the extreme BMI clustering. The residual amount of spatial autocorrelation is similar to what we found when randomizing the BMI values or what we would expect by chance.

Although the regression models were able to account for nearly all of the clustering of extreme BMI residual values, we mapped the remaining observations (<3.45%) that demonstrated persistent clustering. The remaining observations with low/low and high/high BMI residual values had a similar geographic pattern compared to the spatial pattern for the unadjusted BMI values. Throughout all the models, three locations within a 4.8 km (3 mi) radius persisted with 50 or more individuals who had very high BMI values and no locations had 50 or more individuals with low BMI values.

Our analysis is cross-sectional and therefore cannot infer causality. While most neighborhood studies employ cross-sectional designs, longitudinal studies may better capture the fluidity of the neighborhood environment and how changes over time affect health outcomes. Many neighborhoods are fairly stable within the time frame of a few years, although changes in residence could also influence neighborhood characteristics. Our findings that individual characteristics explained much of the variation in BMI, rendered Global Moran’s I to be non-significant and reduced the amount of clustering of the BMI residuals, suggest that individual factors may be the cause of the clustering. Neighborhood selection, whether voluntary or involuntary, is believed to be driven by individual choices, conditions, and preferences in response to life events such as illness, change in job, retirement, or other changes in family composition or social status. These findings support the idea that additional research may be needed to understand individual selection factors that may be correlated with health. Compared with other studies, we had a very large sample size; however, our cohort of adult Kaiser Permanente Northern California (KPNC) patients with diabetes may not be generalizable to the larger population of adults with diabetes, although it may be generalizable to the larger Kaiser population of adults with diabetes in Northern California and also to adults with diabetes who have health insurance. KPNC members make up 30% of the population of Northern California and have similar demographic and socioeconomic distributions to the population from the surrounding geographical region except for the very extremes of the income distribution; fewer very rich and fewer very poor [17]. We did control for population density to reduce the risk that our findings were an artifact of high population urban centers, street connectivity, and walkability [34, 35]. Although we controlled for a number of additional individual and neighborhood factors, we did not have a direct measure of the physical activity environment. A strength of this analysis is that it is a unique population – adults with diabetes – a chronic condition where diet and BMI play an important role in disease management and progression. Our novel approach to assessing a health outcome at the individual point location may be beneficial in identifying areas of greatest clinical and health intervention need. Point level data helps identify locations within towns, ZIP codes, or counties, and may help target the highest risk areas for neighborhood-wide, educational outreach (e.g., educational billboards).

## Conclusions

Our findings indicate a significant level of clustering of extreme high and low BMI values among adults with diabetes across Northern California. Individual demographic and socioeconomic factors accounted for somewhat more (68% vs 50%) than neighborhood or contextual factors, this finding suggests that individual choices, conditions, and preferences may play a strong role in how individuals select the neighborhoods in which they live. Although individual demographic and socioeconomic factors explain much of the BMI clustering, these methods may help identify where people at greatest risk live. While recent studies have found spatial clustering of cardiometabolic risk factors [36–38], confirmatory evidence of the relationship between neighborhood characteristics and spatial clustering of cardiometabolic risk factors is still needed in order to identify the role of community in promoting behavior change [36]. Given the cross-sectional nature of the data, it is unknown whether spatially targeting health educational resources or other interventions to these areas of high clustering would be a cost-effective public health strategy.

## Authors’ information

Barbara A Laraia and Samuel D Blanchard shared first author.

## Electronic supplementary material

Additional file 1: **Literature review of spatial autocorrelation methods in the public health literature.** (DOCX 31 KB)

Additional file 2: Table S1: Examples of spatial cluster methods reported in the public health literature. Literature review table detailing examples of spatial cluster methods reported in the public health literature which includes focal target (spatial unit), cluster algorithm(s), target (attribute), location, and reference. (XLSX 15 KB)
