# Supplementary material for: Spatial pattern of body mass index among adults in the diabetes study of Northern California (DISTANCE)
Source: Int J Health Geogr. 2014 Dec 4;13:48. doi: 10.1186/1476-072X-13-48 (PMC4320620; doi:10.1186/1476-072X-13-48)
Supplement: Supplementary file 1 — Additional file 1: Literature review of spatial autocorrelation methods in the public health literature. (DOCX 31 KB) [file 12942_2014_622_MOESM1_ESM.docx]

**Spatial Autocorrelation Methods in the Public Health Literature**

Some of the most widely used methods in the public health literature for calculating global and local spatial autocorrelation using distance and zonal operators include Kulldorff spatial scan [1], Cuzick-Edwards k-Nearest Neighbor [2], Ripley's K [3], Geary's C [4], Getis-Ord General G, Getis-Ord Gi, Getis-Ord Gi* [5], Global Moran's I, and Local Moran’s I [6, 7]. There are few studies that use points as their unit of analysis, and those that do are assessing the spatial autocorrelation of exposure measures in the physical landscape such as infrastructure, pollution sources [8] or locations of advertisements using Ripley's K [9], or retail stores using Global Moran's I [10]. The majority of the public health literature examining spatial autocorrelation of health outcomes focuses on understanding the pattern of areal units that represent administrative or statistical units, for example assigning a group mean or prevalence ratio to a polygon and using the centroid of the polygon, or in some cases, the adjacent edge, to assess spatial relationships. Examples that examine the spatial pattern of chronic disease health outcomes typically examine disease incidence or health service usage such as using villages to assess prevalence of obesity with Kulldorff spatial scan [11], postal codes to assess obesity, BMI and health behaviors with Global and Local Moran's I [12, 13], and diabetes-related hospital visitation rates with spatial filtering [14]. Additional health outcomes that have been assessed for spatial autocorrelation are incidence of breast cancer with Kulldorff spatial scan and k-Nearest Neighbor [15], tuberculosis with Ripley's K and k-Nearest Neighbor [16], incidence of West Nile virus with Local Moran's I and Kulldorff spatial scan at the county level [17], or Census tracts to assess mortality with Local Moran's I and Local Getis-Ord Gi and Gi* [18]. While the above studies did examine spatial variation in population measures (e.g., incidence, prevalence, averages), this is the first study that we know of that examines spatial variation in individual-level health outcomes. For a detailed table summarizing a selection of spatial clustering methods reported in the public health literature see, Table S1 that accompanies this article.

**References**

1. Kulldorff M: **A spatial scan statistic.** *Communications in Statistics-Theory and Methods* 1997, **26**(6):1481-1496.

2. Cuzick J, Edwards R: **Spatial Clustering for Inhomogeneous Populations.** *Journal of the Royal Statistical Society Series B-Methodological* 1990, **52**(1):73-104.

3. Boots BN, Getis A: *Point Pattern Analysis*: Sage Publications; 1988.

4. Cliff AD, Ord JK: *Spatial autocorrelation*. London,: Pion; 1973.

5. Getis A, Ord JK: **The Analysis of Spatial Association by Use of Distance Statistics.** *Geographical Analysis* 1992, **24**(3):189-206.

6. Moran PAP: **Notes on Continuous Stochastic Phenomena.** *Biometrika* 1950, **37**(1-2):17-23.

7. Anselin L: **Local Indicators of Spatial Association - Lisa.** *Geographical Analysis* 1995, **27**(2):93-115.

8. Fisher JB, Kelly M, Romm J: **Scales of environmental justice: combining GIS and spatial analysis for air toxics in West Oakland, California.** *Health Place* 2006, **12**(4):701-714.

9. Hillier A, Cole BL, Smith TE, Yancey AK, Williams JD, Grier SA, et al. **Clustering of unhealthy outdoor advertisements around child-serving institutions: A comparison of three cities.** *Health Place* 2009, **15**(4):935-945.

10. Sharkey JR, Horel S, Han D, Huber JC, Jr: **Association between neighborhood need and spatial access to food stores and fast food restaurants in neighborhoods of colonias.** *Int J Health Geogr* 2009, **8**:9.

11. Dahly DL, Gordon-Larsen P, Emch M, Borja J, Adair LS: **The spatial distribution of overweight and obesity among a birth cohort of young adult Filipinos (Cebu Philippines, 2005): an application of the Kulldorff spatial scan statistic.** *Nutr Diabetes* 2013, **3**:e80.

12. Mobley LR, Finkelstein EA, Khavjou OA, Will JC: **Spatial analysis of body mass index and smoking behavior among WISEWOMAN participants.** *J Womens Health* 2004, **13**(5):519-528.

13. Schuurman N, Peters PA, Oliver LN: **Are obesity and physical activity clustered? A spatial analysis linked to residential density.** *Obesity* 2009, **17**(12):2202-2209.

14. Curtis AJ, Lee WA: **Spatial patterns of diabetes related health problems for vulnerable populations in Los Angeles.** *Int J Health Geogr* 2010, **9**:43.

15. Meliker JR, Jacquez GM, Goovaerts P, Copeland G, Yassine M: **Spatial cluster analysis of early stage breast cancer: a method for public health practice using cancer registry data.** *Cancer Causes Control* 2009, 20(7):1061-1069.

16. Munch Z, Van Lill SWP, Booysen CN, Zietsman HL, Enarson DA, Beyers N: **Tuberculosis transmission patterns in a high-incidence area: a spatial analysis.** *Intl J Tuberculosis Lung Disease* 2003, **7**(3):271-277.

17. Sugumaran R, Larson SR, Degroote JP: **Spatio-temporal cluster analysis of county-based human West Nile virus incidence in the continental United States.** *Int J Health Geogr* 2009, **8**:43.

18. Burra T, Jerrett M, Burnett RT, Anderson M: **Conceptual and practical issues in the detection of local disease clusters: a study of mortality in Hamilton, Ontario.** *Canadian Geographer-Geographe Canadien* 2002, **46**(2):160-171.

**Additional References in Table 1S**

Apparicio P, CloutierM, Shearmur R: **The case of Montréal's missing food deserts: Evaluation of accessibility to food supermarkets.** *Intl J Health Geogr* 2007, **6**(4).

Assuncao RM, Reis EA: **A new proposal to adjust Moran’s I for population density.** *Statistics in Medicine* 1999, **18**: 2147–2162.

Austin SB, Melly SJ, Sanchez BN, Patel A, Buka S, Gortmaker SL: **Clustering of fast-food restaurants around schools: A novel application of spatial statistics to the study of food environments.** *Am J Public Health* 2005, **95**(9):1575-1581.

Bastin L, Rollason J, Hilton A, Pillay D, Corcoran C, Elgy J, Lambert P, De P, Worthington T, Burrows K: **Spatial aspects of MRSA epidemiology: a case study using stochastic simulation, kernel estimation and SaTScan.** *Intl J Geographical Information Science* 2007, **21**(7): 811-836.

Chen Y, Yi Q, Mao Y: **Cluster of liver cancer and immigration: A geographic analysis of incidence data for Ontario 1998–2002.** *Intl J Health Geogr* 2008, **7**(28).

Fang L, Yan L, Liang S, de Vlas SJ, Feng D, Han X, Zhao W, Xu B, Bian L, Yang H, Gong P, Richardus JH, Cao W: **Spatial analysis of hemorrhagic fever with renal syndrome in China.** *BMC Infectious Diseases* 2006, **6**(77).

García-Alonso CR, Salvador-Carulla L, Negrín-Hernández MA, Moreno-Küstner B: **Development of a new spatial analysis tool in mental health: Identification of highly autocorrelated areas (hotspots) of schizophrenia using a Multiobjective Evolutionary Algorithm model (MOEA/HS).** *Epidemiologia e Psichiatria Sociale* 2010, **19**: 302-313.

Glavanakov S, White DJ, Caraco T, Lapenis A, Robinson, GR, Szymanski BK, Maniatty WA: **Lyme disease in New York State: Spatial pattern at a regional scale.** *The American Society of Tropical Medicine and Hygiene* 2001, **65**(5):538–545.

Green C, Hopp RD, Young TK, Blanchard JF: **Geographic analysis of diabetes prevalence in an urban area.** *Soc Sci Med* 2003, **57**:551–560.

Goovaerts P, Jacquez GM: **Detection of temporal changes in the spatial distribution of cancer rates using local Moran’s I and geostatistically simulated spatial neutral models.** *J Geographical Systems* 2005, **7**:137–159.

Haque U, Scott LM, Hashizume M, Fisher E, Haque R, Yamamoto T, Glass GE: **Modelling malaria treatment practices in Bangladesh using spatial statistics.** *Malaria Journal* 2012, **11**(63).

Hinman SE, Blackburn JK, Curtis A: **Spatial and temporal structure of typhoid outbreaks in Washington, D.C., 1906–1909: evaluating local clustering with the *Gi** statistic.** *Intl J Health Geogr* 2006, **5**(13).

Hinrichsen VL, Klassen AC, Song C, Kulldorff M: **Evaluation of the performance of tests for spatial randomness on prostate cancer data.** *Intl J Health Geogr* 2009, **8**(41).

Khormi HM, Kumar L, Elzahrany RA: **Modeling spatio-temporal risk changes in the incidence of dengue fever in Saudi Arabia: a geographical information system case study.** *Geospatial Health* 2011, **6**(1):77-84.

Klassen AC, Kulldorff M, Curriero F: **Geographical clustering of prostate cancer grade and stage at diagnosis, before and after adjustment for risk factors.** *Intl J Health Geogr* 2005, **4**(1).

Lin G, Zhang T: **Loglinear residual rests of Moran’s I autocorrelation and their applications to Kentucky breast cancer data.** *Geographical Analysis* 2007, **39**: 293–310.

Liu Y, Li X, Wang W, Li Z, Hou M, He Y, Wu W, Wang H, Liang H, Guo X: **Investigation of space-time clusters and geospatial hot spots for the occurrence of tuberculosis in Beijing.** *Intl J Tuberculosis Lung Disease* 2012, **16**(4):486–491.

López-Cevallos DF, Chi C: **Assessing the context of health care utilization in Ecuador: A spatial and multilevel analysis.** *BMC Health Services Research* 2010, **10**(64).

Lorant V, Thomas I, Deliege D, Tonglet R: **Deprivation and mortality: the implications of spatial autocorrelation for health resources allocation.** *Soc Sci Med* 2001, **53**: 1711–1719.

Loughnan ME, Nicholls N, Tapper NJ: **Demographic, seasonal, and spatial differences in acute myocardial infarction admissions to hospital in Melbourne Australia.** *Intl J Health Geogr* 2008, **7**(42).

Maciel ELN, Pan W, Dietze R, Peres RL, Vinhas SA, Ribeiro FK, Palaci M, Rodrigues RR, Zandonade E, Golub JE: **Spatial patterns of pulmonary tuberculosis incidence and their relationship to socio-economic status in Vitoria, Brazil.** *Intl J Tuberculosis and Lung Disease* 2010, **14**(11):1395–1402.

Malczewski J: **Exploring spatial autocorrelation of life expectancy in Poland with global and local statistics.** *GeoJournal* 2010, **75**:79–92.

Mandal R, St-Hilaire S, Kie JG, Derryberry D: **Spatial trends of breast and prostate cancers in the United States between 2000 and 2005.** *Intl J Health Geogr* 2009, **8**(53).

Michel P, St-Onge L, Lowe A, Bigras-Poulin M, Brassard P: **Geographical variation of Crohn's disease residual incidence in the Province of Quebec, Canada.** *Intl J Health Geogr* 2010, **9**(22).

Mitra R, Buliung RN, Faulkner GEJ: **Spatial clustering and the temporal mobility of walking school trips in the Greater Toronto Area, Canada.** *Health Place* 2010, **16**:646–655.

Ngowi HA, Kassuku AA, Carabin H, Mlangwa JE, Mlozi MR, Mbilinyi BP, Willingham AL 3rd: **Spatial clustering of Porcine Cysticercosis in Mbulu District, Northern Tanzania.** *PLoS Neglected Tropical Diseases* 2010, **4**(4): e652.

Osei FB, Duker AA: **Spatial and demographic patterns of Cholera in Ashanti region – Ghana.** *Intl J Health Geogr* 2008, **7**(44).

Pouliou, T, Elliott SJ: **An exploratory spatial analysis of overweight and obesity in Canada.** *Prev Med* 2009, **48**:362–367.

Rojas F: **Poverty determinants of acute respiratory infections among Mapuche indigenous peoples in Chile's Ninth Region of Araucania, using GIS and spatial statistics to identify health disparities.** *Intl J Health Geogr* 2007, **6**(26).

Ross A, Davis S: **Point pattern analysis of the spatial proximity of residences prior to diagnosis of persons with Hodgkin's Disease.** *Am J Epidemiology* 1990, **132**(11990): S53-S62.

Ruiz-Moreno D, Pascual M, Emch M, Yunus M: **Spatial clustering in the spatio-temporal dynamics of endemic cholera.** *BMC Infectious Diseases* 2010, **10**(51).

Saxena R, Nagpal BN, Das MK, Srivastava A, Gupta SK, Kumar A, Jeyaseelan AT, Baraik VK: **A spatial statistical approach to analyze malaria situation at micro level for priority control in Ranchi district, Jharkhand.** *Indian J Med Research* 2012, **136**: 776-782.

Stoler J, Weeks JR, Getis A, Hill AG: **Distance threshold for the effect of urban agriculture on elevated self-reported malaria prevalence in Accra, Ghana.** *Am J Trop Med Hyg* 2009, **80**(4): 547–554.

Tsai, P, Lin M, Chu C, Perng C: **Spatial autocorrelation analysis of health care hotspots in Taiwan in 2006.** *BMC Public Health* 2009, **9**(464).

Uthman OA, Yahaya I, Ashfaq K, Uthman MB: **A trend analysis and sub-regional distribution in number of people living with HIV and dying with TB in Africa, 1991 to 2006.** *Intl J Health Geogr* 2009, **8**(65).

Wu J, Wang J, Meng B, Chen G, Pang L, Song X, Zhang K, Zhang T, Zheng X: **Exploratory spatial data analysis for the identification of risk factors to birth defects.** *BMC Public Health* 2004, **4**(23).

Yiannakoulias N: **Synthesizing waterborne infection prevalence for comparative analysis of cluster detection.** *Geospatial Analysis of Environmental Health* 2011, **4**:457-472.

Zhanga C, Luob L, Xub W, Ledwitha V. **Use of local Moran's I and GIS to identify pollution hotspots of Pb in urban soils of Galway, Ireland**. *Science of the Total Environment* 2008, **398**: 212-221.
